# Characterisation of the BOLD response time course at different levels of the auditory pathway in non-human primates

**DOI:** 10.1016/j.neuroimage.2009.12.103

**Published:** 2010-04-15

**Authors:** Simon Baumann, Timothy D. Griffiths, Adrian Rees, David Hunter, Li Sun, Alexander Thiele

**Affiliations:** Institute of Neuroscience, Newcastle University, Framlington Place, Newcastle upon Tyne, NE2 4HH, UK

## Abstract

Non-human-primate fMRI is becoming increasingly recognised as the missing link between the widely applied methods of human imaging and intracortical animal electrophysiology. A crucial requirement for the optimal application of this method is the precise knowledge of the time course of the Blood Oxygenation Level Dependent (BOLD) signal. We mapped the BOLD signal time course in the inferior colliculus (IC), medial geniculate body (MGB) and in tonotopically defined fields in the auditory cortex of two macaques. The results show little differences in the BOLD-signal time courses within the auditory pathway. However, we observed systematic differences in the magnitude of the change in the BOLD signal with significantly stronger signal changes in field A1 of the auditory cortex compared to field R. The measured time course of the signal was in good agreement with similar studies in human auditory cortex but showed considerable differences to data reported from macaque visual cortex. Consistent with the studies in humans we measured a peak in the BOLD response around 4 s after the onset of 2-s broadband noise stimuli while previous studies recorded from the primary visual cortex of the same species reported the earliest peaks to short visual stimuli several seconds later. The comparison of our results with previous studies does not support differences in haemodynamic responses within the auditory system between human and non-human primates. Furthermore, the data will aid optimal design of future auditory fMRI studies in non-human primates.

## Introduction

Imaging in non-human primates is becoming a valuable set of tools to bridge the traditional gap between invasive animal electrophysiology and human imaging. Auditory imaging studies can inform understanding of the basic organisation of the auditory cortex (Kayser et al., 2005; Petkov et al., 2006; Tanji et al., 2010), as well as higher cognitive functions such as perception and control of vocalisations (Gil-da-Costa et al., 2004; Petkov et al., 2008). The latter two studies demonstrate the potential of the technique to establish the phylogenetic development of high-level cognition as well as fundamental aspects of perception. While the systematic application of imaging to non-human primates is relatively new, the field can rely on more than a decade of experience in human imaging. However, there are a number of basic BOLD response properties that require cross-validation between species to ensure that the interpretation of data is not based on invalid assumptions.

Functional Magnetic Resonance Imaging (fMRI), the most widely used method in human and non-human primate imaging, relies on a relatively precise knowledge of the time course of the Blood Oxygen Level Dependent (BOLD) signal to optimally model neural activity. While the few studies that report detailed time courses of the BOLD signal in monkeys are mostly confined to the visual cortex, there are no such data at high temporal resolution available for the auditory system in non-human primate. Moreover, few studies of the human auditory system provide an optimal reference for comparison. To obtain data from the auditory system, unbiased by the high-volume noise generated during recording of the BOLD signal, special recording designs such as “sparse temporal sampling” are required. In a “sparse” design, the auditory stimulus is presented in a period undisturbed by scanner noise which is followed by a short period of recording where typically the peak of the delayed BOLD response to the sound is captured. Information on the time course can only be obtained by repeatedly shifting the onset of the auditory stimulus relative to the recording period in order to capture different time points of the BOLD time course in the process. Using this method, Belin et al. (1999) characterised the time course of the BOLD response in response to complex harmonic sound stimuli in the human auditory cortex. In good agreement with a later, similar study in humans (Backes and van Dijk, 2002) a peak in the BOLD response was demonstrated 3 s after sound onset in the primary auditory cortex and 4 s in higher auditory areas for brief sound stimuli. These results differ considerably from studies in the primary visual cortex of monkeys (Logothetis, 2002; Logothetis et al., 1999, 2001) in which the BOLD response peaks at 7–10 s after onset of brief stimuli and with a plateau in the same time range after sustained visual stimulation of 10–20 s.

Differences of more than 4 s in the temporal pattern of the BOLD response in two primary sensory areas cannot be explained by differences in the time course of the underlying neuronal activity with latencies in the order of milliseconds. Discrepancies of such magnitudes can only be explained either by differences in the underlying haemodynamics of the BOLD signal between different areas, or species, or by methodological differences in BOLD signal measurement, and the state of the subject (awake, under anaesthesia). Such uncertainties are a serious challenge for the optimal design of auditory fMRI experiments in monkeys. Independent of whether the aim is to model the haemodynamic response in a continuous design or to capture the BOLD response peak in a “sparse” design, a misjudgement of the BOLD time course by 4 s or more, would lead to a considerable error in the estimation of the response. Furthermore, if verified, large differences in the haemodynamics between areas could pose problems for the comparison of activity between these areas.

Here we characterise the time course of the BOLD response in multiple cortical and subcortical areas of the auditory system of the macaque monkey to short and sustained sound stimuli. In addition to providing a basis for the design of future auditory fMRI studies in non-human primates, we aimed to address the following questions: is there a species-specific difference in the time course of haemodynamic responses between humans and macaques measured under comparable conditions; do data obtained from the monkey auditory system better match data reported from visual system of the same species, and do considerable differences exist in the time course of the BOLD signal between auditory areas that would complicate the comparison of activity within the auditory pathway?

## Materials and methods

### Animals

The data were obtained from 20 fMRI scanning sessions with two male macaque monkeys (Macaca mulatta) weighting 9 and 13 kg (10 sessions per animal). Both animals (monkey C and monkey W) were previously habituated to the scanner environment and were scanned in the awake and resting state. A primate chair was used to position the animal in the magnet. During the recording, the head of the animal was positioned with a custom-made polyetheretherketone (PEEK) head holder attached to a cranial implant. Details of the surgical procedures and the materials used for the cranial implant are given in Thiele et al. (2006). All experiments were carried out in accordance with the UK Animals (Scientific Procedures) Act (1986), European Communities Council Directive 1986 (86/609/EEC) and the US National Institutes of Health Guidelines for the Care and Use of Animals for Experimental Procedures and were performed with great care to ensure the well-being of the animals.

### Sound stimuli and presentation

Sound stimuli were created using custom-made scripts in MATLAB 7.1 (MathWorks, Natick, USA) with sample rate of 44.1 kHz and 16-bit resolution. The stimuli for characterising the time course of the BOLD signal were based on a fixed amplitude, random-phase noise carrier with a passband between 25 Hz and 16 kHz. The carriers were amplitude modulated with a sinusoidal envelope of 100% depth at a frequency of 10 Hz. These sounds were designed to ensure maximal excitation in a wide range of cortical and subcortical auditory areas (Joris et al., 2004). In the MRI scanner, we presented the sound stimuli at two different durations (2 s, 8 s) at an rms sound pressure level (SPL) of 85 dB. Sound was delivered through loudspeakers, which were positioned above the rf-shielded top opening of the scanner.

In order to map tonotopic fields in each monkey, we used amplitude modulated bandpass noise with three different passbands: 0.5–1 kHz, 2–4 kHz and 8–16 kHz. The bandpass stimuli were amplitude modulated applying the same parameters as described in the previous paragraph. The duration of the bandpass stimuli was 9 s (filling the silent interval between two subsequent volume acquisitions completely). The tonotopic mapping data were obtained at an rms sound level of 75 dB SPL using custom adapted electrostatic headphones based on a Nordic Neurolab system (NordicNeuroLab, Bergen, Norway). Stimuli were presented and synchronised with the data recording using Cortex software (NIMH, http://www.cortex.salk.edu). The SPL of the stimuli was verified in the scanner with an MR-compatible free-field condenser microphone connected by an extension cable to a Bruel&Kjaer Type 2260 sound level meter (all Bruel&Kjaer, Naerum, Denmark).

### MRI hardware and imaging

Data were recorded using an actively shielded, vertical 4.7 T MRI scanner (Bruker Biospec 47/60 VAS) equipped with a Bruker GA-38S gradient system that has an inner-bore width of 38 cm (Bruker Medical, Ettlingen, Germany). To achieve optimal signal to noise ratios we employed a custom-made RF-surface coil. This circular shaped transceiver coil (7 cm inner diameter) was placed over the right temporal lobe using a custom made coil holder that was freely adjustable in three axes. The circular, open shape allowed positioning around the ear without covering the ear canals. The application of a surface coil with a limited field of view restricts the recorded data to the right hemisphere. However, based on previous haemodynamic response studies in humans (Backes and van Dijk, 2002; Belin et al., 1999) we do not expect differences in the BOLD response time course between the hemispheres.

The area covered by the MRI scans encompassed the entire temporal lobe and also the MGB and the ICs in the ascending auditory pathway. Sixteen slices of 1.5-mm thickness with no gap were oriented parallel to the lateral sulcus to obtain a plane of activity corresponding to the auditory cortex. The exact position was chosen with the help of a sagittal MDEFT (Modified Driven Equilibrium Fourier Transform; Lee et al., 1995) structural scan (detailed parameters same as with structural scans below). The approximate position of the field of view (FOV) is visualised in Fig. 2B.

Functional data were acquired using single-shot gradient-recalled echo-planar imaging sequences (GE-EPI; Mansfield, 1977) with the following parameters: TE: 21 ms, volume acquisition time (TA) 1 s, flip angle (FA): 90°, spectral bandwidth: 200 kHz, FOV: 12.8 × 9.6 cm^2^, on a grid of 128 × 96 voxels resulting in a resolution of 1 × 1 mm^2^ in-plane resolution. The separation of volume acquisitions (TR) depended on the experiment; it was 18 s for the experiment characterizing the HRF and 10 s for the tonotopic mapping experiment. Each volume acquisition was initiated by an external trigger provided by the presentation software.

Structural images (T1-weighted) used the same slice geometry as the functional scans of the same session to simplify coregistration. The imaging parameters of the MDEFT sequence were TE: 6 ms, TR: 1890 ms, FA: 19.7°, FOV 12.8 × 9.6 cm^2^ using an encoding matrix of 256 × 256 to give an in-plane resolution of 0.5 × 0.375 mm^2^ per voxel. Correct overlap of structural and functional images has been rechecked manually.

**Fig. 1:**
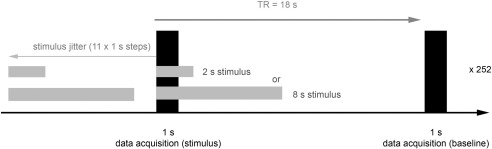
Schema of the ‘sparsed’-fMRI design with stimulus jitter. Two sound stimuli of different durations were presented in separate sessions. The onsets of the stimuli preceded the data acquisition by different time periods from 0 to 11 s to sample the time course of the BOLD response. Data acquisitions with stimulus presentation were interleaved with acquisitions without stimulus to obtain baseline trials.

To avoid interaction of the stimulus-induced BOLD responses with the response evoked by the gradient noise of the scanner we applied a ‘sparse-sampling’ paradigm. For the haemodynamic response experiment, images were acquired every 18 s while the acquisition of one volume was completed within 1 s. In 12 conditions, the onsets of the stimuli were positioned at different time points from 0 to 11 s before image acquisition to obtain a sampling of the BOLD response at a 1 s resolution from 0 to 11s after sound onset (Fig. 1). The repetition time of 18 s between acquisitions was designed to ensure that the haemodynamic response to the stimuli fell to baseline levels for all conditions before acquisition of the subsequent volume. This requirement was tested in pilot experiments using a repetition time of 25 s and time points sampled every 2 s. Trials with and without stimulus presentation were alternated within blocks which permitted baseline activity to be measured during periods of silence. A total of 504 acquisition volumes per session resulted in 252 baseline and 252 stimulus volumes, with 21 volumes per time point to characterise the haemodynamic response function. Altogether, we recorded data in five sessions per animal for each stimulus duration. With two animals and two stimulus-duration conditions this resulted in 20 sessions in total.

For the tonotopic mapping of auditory fields we used a sparse-sampling design with 10 s separation between volume acquisitions (TR = 10 s). The bandpass stimuli were presented during the first 8 s preceding the acquisition and during the volume acquisition itself. Again, trials with and without stimulus presentation were alternated to obtain an equal number of volumes with sound presentation and silent baseline data. For each animal, a tontopic map was acquired in one session of 720 volume acquisitions, resulting in 120 volumes for each of the three frequency bandwidths.

### Data analysis

For preprocessing and general linear model analysis we employed SPM5 implemented in Matlab 7.1. Image volumes from each session were realigned to the first volume before smoothing the data with a kernel of 2 mm full-width half-maximum. Functional *t*-value maps were computed for each condition (time points of haemodynamic response or frequency bandwidth, respectively) by fitting a general linear model in the form of a boxcar function for stimulus versus baseline volumes. We adjusted the time series that went into the model for global signal fluctuations (global scaling) and applied a high pass filter with a cut-off of 300 s to account for slow signal drifts. Because of the long repetition times of the sparse-sampling paradigm, corrections for serial correlations and low-pass filtering of the time series were not applied.

In addition to the regressors for the conditions that modelled the different time points, six movement parameters derived from the realignment process (derivatives of movement vectors) were implemented in the model as regressors of no interest to account for movement artefacts.

### Identification of functional fields in the auditory cortex

In order to identify distinct functional auditory fields in the superior temporal plane we calculated tonotopic gradients based on the differential responses to the different frequency ranges of the bandpassed noise stimuli (see Petkov et al., 2006, for a detailed description of the definitions of the auditory fields). The resulting field borders were subsequently superimposed onto the functional t-maps in order to assign the observed maxima of activity to auditory fields.

### BOLD response time course

**Fig. 2:**
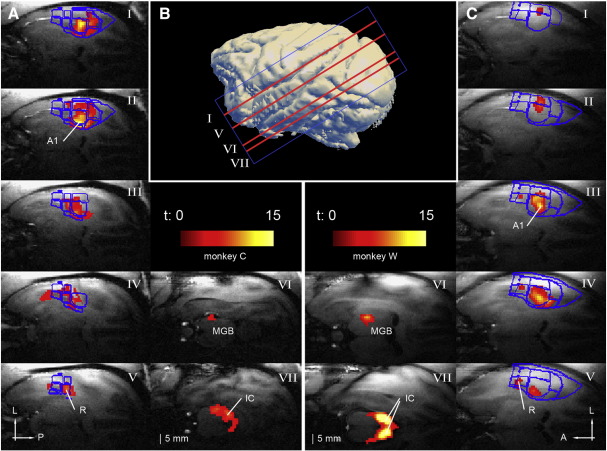
T-maps of the broadband noise vs. silence contrast for the two monkeys. T-maps are displayed in panel A for monkey C and in panel C for monkey W. The approximate location of the displayed slices I–VII is shown in panel B. Borders of the individually identified auditory fields are outlined in blue. For a detailed organisation of the fields see Fig. 3. A1: primary auditory cortex, R: rostral auditory field, MGB: medial geniculate body, IC: inferior colliculus, A: anterior, P: posterior, L: lateral.

The data for the BOLD response time course analysis were obtained by extracting the raw data time series from regions of interest (ROIs) located in fields A1 and R in the auditory cortex and the subcortical structures of the inferior colliculus (IC) and the medial geniculate bodies (MGB), all in the right hemisphere. The ROIs consisted of voxels within a sphere of 1 mm radius centred on the *t*-value maximum derived from the “broad band stimulus vs. silence” contrast in each brainstem structure or cortical field. Pilot experiments established that the chosen radius showed the lowest variance of the BOLD response within a session. The brainstem structures were identified based on the structural scan and contained a single functional maximum within the anatomically defined area. Three maxima were consistently observed in the auditory cortex of both monkeys. Two were located in the tonotopically defined field A1 and a third in the field R (see Fig. 2). Of the two maxima in A1 we chose the more medial one for the ROIs because it showed consistently higher *t*-values.

We calculated the time course of the percent signal change for each stimulus duration and animal by subtracting the mean baseline signal of the session from the mean of the BOLD response at each time point and dividing it by the baseline signal. *t*-tests were performed on the resulting time courses from the different areas. Response peaks were identified for each session of the 2-s duration stimulus condition and *t*-tests were performed for the latency and magnitudes between the peaks in the different areas.

The linear transforms of the mean responses to the 2-s stimuli were calculated to predict the responses to the 8-s stimuli based on an assumption of proportionality in the local average of the neuronal responses over a period of time (Boynton et al., 1996). The predicted response for the 8-s stimuli was modelled by a linear combination of four 2-s response time courses with a time delay of 2 s from time-course to time-course.

## Results

The initial aim of this study was to find MRI parameters that allow us to record the haemodynamic response at multiple levels of the macaque auditory system from the IC up to the auditory cortex. Circular, open surface coils that we designed for this purpose were arranged around the ear of the monkey. In combination with a field strength of 4.7 T, the coils provided the means to obtain a high signal-to-noise ratio of 100–200 in the entire temporal lobe. In addition, we applied a sparse design with a relatively long TR of 18 s which ensured that we compared the sound delivery conditions to conditions when no sound was delivered. As a result of these optimisations we were able to record robust blood oxygenation level dependent (BOLD) responses in cortical and subcortical areas of the auditory system at an in-plane resolution of 1 × 1 mm^2^.

### Activation map for broadband noise versus silence

For amplitude-modulated random-phase broadband noise compared to silence we found significant BOLD responses in the IC, the MGB, and in the entire core and some of the belt areas of the auditory cortex. The corresponding t-maps obtained from the monkeys are shown in Fig. 2. A detailed list of t-maxima for each session are summarised in Table 1. Although not characterised in detail in this study, areas of significant activity extended continuously from the IC into the nucleus of the lateral lemniscus and along the inferior brachium into the MGB (partly visible in Fig. 2, panel A VII and C VII). Significant BOLD responses were observed in all sessions in the auditory areas of the right hemisphere over which the surface coil was placed (see also Table 1). However, the left IC was often significantly activated as well. In some cases the left MGB and even the left auditory cortex showed significant BOLD responses despite the relatively low signal to noise ratio in these areas due to the large distance from the surface coil. The maximal *t*-values after 2-s sound stimulations or the plateau of the *t*-values after 8-s stimulation were reached at approximately 4 s after sound onset. The maximum of the t-map in the auditory cortex was localised in the posterior third at the most medial corner of the superior temporal plane (Fig. 2, panel A II and C III). This maximum was later identified as being part of the field A1. A second local maximum was usually found a few millimetres lateral of the first, midway between the medial and the lateral border of the superior temporal plane. A third local maximum with slightly lower *t*-value, later identified as being localised in field R, was observed about half a centimetre more anterior of the previously described maxima. The pattern of these three maxima was consistent across the sessions and occurred with slight variations in both monkeys.

### Identification of auditory fields based on tonotopy

We identified individual functional fields in the auditory cortex based on previous studies in macaques showing that reversing tonotopic gradients in anterior-posterior direction co-localise with microanatomically distinguishable areas (Hackett et al., 1998; Kaas and Hackett, 2000; Kaas et al., 1999; Petkov et al., 2006). In particular the anterior–posterior border at the frequency reversal between the cortical fields A1 and R and to a lesser extent between R and RT, and A1 and the caudal fields CL and CM are well established. According to these studies the tonotopic gradient runs from high frequencies at the posterior border to low frequencies at the anterior border of A1, field R shows a reversed gradient running from low to high and the field RT features again the same gradient direction as A1. Similar, parallel tonotopic gradients in the lateral belt areas and an additional reversal in the caudal belt have been reported (Rauschecker et al., 1995).

**Fig. 3:**
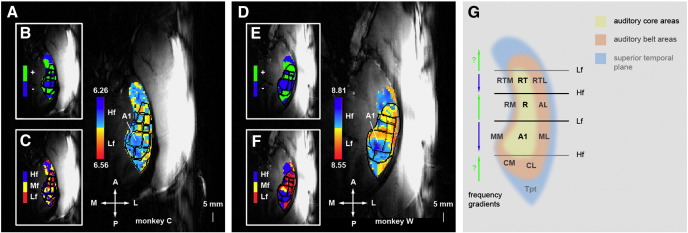
Tonotopic organisation of the auditory cortex. Panels A and D display the *t*-value difference map for high frequency bandpass noise (Hf) compared to low frequency bandpass noise (Lf) in the two monkeys C and W. A map of the voxels that give the strongest response to one of three frequency bands Hf, Mf and Lf is displayed in panels C and F. Panels B and E show the location of mirror reversed tonotopic gradients (positive: low to high from posterior to anterior; negative: high to low from posterior to anterior. The borders of the identified fields are outlined in black. G shows the organisation of auditory fields according to Hackett et al., 2001 and Petkov et al., 2006. Hf: high frequency band (8–16 kHz), Mf: middle frequency band (2-4 kHz), Lf: low frequency band (0.5–1 kHz), A1: primary auditory cortex, R: rostral field, RT: rostrotemporal field, CM: caudomedial field, CL: caudolateral field, MM: middle medial field, ML: middle lateral field, RM: rostromedial field, AL: anterolateral field, RTM: rostrotemporomedial field, RTL: rostrotemporolateral field. Tpt: temporoparietal junction.

First inspection of the data derived from our experiment with three frequency bands of 0.5–1, 2–4 and 8–16 kHz revealed a clear high-to-low gradient in the posterior to anterior direction at the approximate location of A1, with a clear reversal at the lower end in both monkeys (Fig. 3). A detailed analysis on the basis of the methods described in Petkov et al. (2006) revealed the three auditory core fields A1, R and RT, and a number of auditory belt fields. The data from monkey W show a clear extension of the gradients in the core fields into adjacent belt areas. The gradients seem to break down in the area of the lateral convexity where we would expect to find the border with the parabelt fields. Interestingly, low frequency responses predominate in these areas. This leads to a lateral-to-medial gradient in the most posterior area (Tpt) of the superior temporal plane in both animals. Monkey C shows generally similar results, but with less clear gradients particularly in anterior belt areas. Furthermore, the middle frequency band is very well represented in Monkey C (Fig. 3C) while areas that respond best to this band in monkey W are rather underrepresented (Fig. 3F). A further interesting observation in the two animals (and confirmed in other animals not used in this study) concerns the relationship between structure and function. The slight protuberance marking the area where the flat part of the posterior auditory cortex turns into a downward slope towards the anterior auditory cortex predicted very consistently the low frequency area between the fields A1 and R.

As described in Petkov et al. (2006), we can use the identified gradients to delineate the borders of the three core fields A1, R and RT. In addition, we can identify the approximate location of the different belt areas partly based on the same gradients and partly based on their relative position to the identified core areas. The outer borders of the belt areas and area Tpt are mostly defined by the anatomical borders of the superior temporal plane, identifiable from the structural MRI data. The individually identified areas can now be overlaid on the previously established functional t-maps.

Contour lines of the identified auditory fields were overlaid onto the t-maps of the noise vs. silence contrast (Fig. 2). The established field borders show that the maxima of the t-maps fall into the core fields A1 and R. In addition we find occasionally significant activation in the most anterior core field RT, the two belt fields ML and MM lateral and medial from A1, and also in the caudal fields CL and CM. In a next step, we obtained time series from regions of interest (ROI) centred on the maxima of the cortical fields A1 and R that showed reliably significant maxima, and the subcortical areas MGB and IC for detailed time course analysis.

### BOLD response time course

**Fig. 4:**
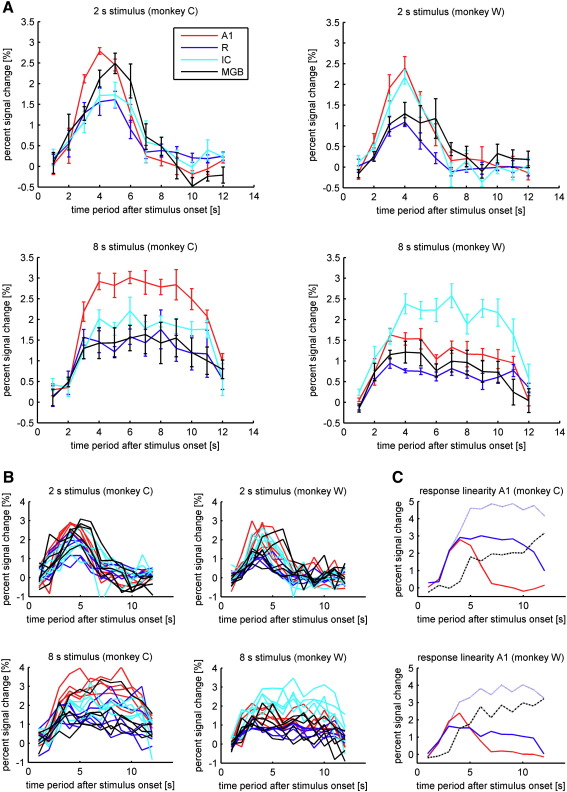
BOLD response time courses for short and sustained stimuli from four different areas in the auditory pathway. A: top row shows average responses to 2-s stimuli; bottom row shows responses to 8-s stimuli. First column: monkey C, second column: monkey W. Bars represent standard error. B: time courses of the individual sessions. Same arrangement as in A. C: measured BOLD signal time-course from A1 after 2-s stimulus (red, solid line), 8-s stimulus (blue, solid line), the prediction of the 8-s stimulus response based on the 2 s data (blue dotted line) and difference between measured and predicted time course (black line).

Systematic variation in the timing of the sound stimuli relative to the volume acquisition provided data for 12 independently measured time points of the BOLD response ranging between 1 and 12 s after the stimulus onset. The average signal for each time point consisting of data from 21 volume acquisitions per session was contrasted to the average signal in the baseline without stimulus presentation. Five sessions were recorded for each of the two monkeys and for the two stimulus durations. The percent change in the signal over the time courses of the BOLD responses in the measured areas are displayed in Fig. 4 for the average of the five sessions (Fig. 4A) and for the individual sessions (Fig. 4B). In all cases the average time courses showed maxima (for 2-s stimulation) or plateaux (for the 8-s stimulation) between the third and the fifth second after sound onset (see Table 2). In general, the signals for the four reported auditory areas show similar time courses. The MGB tended to show slightly later response peaks (significant only for MGB vs. A1, *p* = 0.039) after a similar time course at onset. The average BOLD responses measured in field A1 show, in all but one case, the highest percent signal change at 2.5–3%. Only in animal W for the 8-s stimulation does this value fall below the percent signal change in the IC. In all other condition the time periods 3 and 4 s after sound onset were significantly higher in A1 than in any other area. The values of percent signal change from the other cortical field, R, ranged between 1% and 1.5%, about half of that for A1. The difference between the two cortical areas was consistently significant in both monkeys for the time period 3–5 s after the 2-s stimulus onset and 4–7 s after the 8-s stimulus onset. The percent signal change for the subcortical areas MGB and IC range between the values for the two cortical fields. A comparison of the time courses and magnitudes of the BOLD responses from the different areas show very similar behaviours between the two animals. Only the magnitudes of the BOLD responses from the IC in animal W were consistently higher than in animal C. Yet, the responses in A1 after the long stimulus were relatively weak in the same animal.

Fig. 4C shows the results from an examination of BOLD response linearity based on data from field A1 in both monkeys. These data are representative of similar data in the other measured brain structures. The results show that the measured 8-s BOLD response (solid blue) has a lower amplitude and a slightly shorter plateau onset latency than predicted (dotted blue) by a linear transform of that from the 2-s stimulus (red).

## Discussion

This study demonstrates robust BOLD responses to broadband-noise stimuli mapped with high spatial-resolution at multiple levels of the auditory pathway in awake macaque monkeys. The measured structures include the IC, the MGB and several fields in the auditory cortex. The applied “jittered sparse” paradigm provides detailed haemodynamic response time courses in the imaged areas that can be compared to results from other modalities in macaques and to data from the auditory system in humans. In the following paragraphs, we discuss the measured pattern of activity and compare the time courses in different regions of the auditory system. Furthermore, we contrast our results to data from the visual system in macaques (Logothetis, 2002; Logothetis et al., 2001) and to data from similar studies in the human auditory cortex (Backes and van Dijk, 2002; Belin et al., 1999). Based on these comparisons we discuss how the time course and magnitude of the haemodynamic response depends on the selected areas, species and methodological parameters.

### Activation in subcortical structures

Amplitude-modulated broadband noise is known for its potential to elicit robust responses in a wide range of auditory areas (for review, see Joris et al., 2004). The presented results demonstrate that with the right combination of methods and an optimisation of imaging parameters, responses to these stimuli can be measured with fMRI in several cortical and subcortical areas of non-human primates. While previous studies in non-human primates already reported fMRI activity to a range of sound stimuli in the auditory cortex, auditory activation in subcortical structures of non-human primates has not been demonstrated so far. Even with the more established human fMRI procedures, additional measures such as cardiac gating are usually required to show clear activation in these structures (Backes and van Dijk, 2002; Griffiths et al., 2001; Guimaraes et al., 1998; Harms and Melcher, 2002). In the current study we found reliable responses in the IC and the MGB. BOLD signal changes in the IC were particularly robust and came close to, or sometimes even exceeded, the maximal *t*-values observed in the cortex (see Table 1).

A number of factors may contribute to this result. The generally higher field strengths of monkey dedicated fMRI systems and higher signal-to-noise ratios provided by surface coils are necessary to counterbalance the smaller brain size of monkeys compared to humans and hence higher required resolution to show the same details in cortical areas. While it is mainly the cortex that contributes to the 2- to 3-fold size difference in each dimension, and hence the cube of this number in volume and signal strength difference, subcortical structures such as the IC are not considerably different in size in macaques compared to humans. Thus, the latter structures are expected to profit most in terms of signal increase from the expected higher signal-to-noise ratio due to the high magnetic field strength and the surface coils applied in our experiment. A further advantage of this preparation compared to a standard human setup is the head fixation of the animals to minimise movements. This is particularly relevant because, due to their small size, subcortical structures are more vulnerable to small movements than larger structures. The reason for the higher movement sensitivity is that smaller structures show less overlap in consecutive scanning volumes than larger ones when movements occur between volume acquisitions.

The relatively unreliable BOLD response in the MGB stands in stark contrast to the very robust signal we observed in the IC. We found differences between the two structures in the *t*-values, the number of significantly activated voxels, and particularly in one animal, in the magnitude of the BOLD signal (Figs. 2 and 4, Table 1). We also observed the same pattern of differences in previous experiments with different sound stimuli and other monkeys (not reported here). There are several possible explanations for the observed differences between the two subcortical auditory structures. The source of these differences could either be an artefact of the imaging procedures, physiological differences in the vascularisation, a genuine difference in the neuronal response to auditory stimuli, or a combination of these factors. In the next two paragraphs we briefly discuss the evidence for these possibilities in more detail.

Differences in the size of an activated structure in combination with the dimension of spatial sampling in terms of voxel and smoothing kernel sizes can affect the robustness of activation due movement effects (see discussion above) and due to dilution by not activated areas within the sampled unit. Thus, the slightly larger size of the IC possibly contributes to the measured difference in BOLD response robustness (see discussion above), but it can hardly explain the full extent of the considerable difference we see in some animals. Tests with different smoothing kernels (not shown) demonstrated that the chosen values were at an optimum in terms of robustness. However, there is good evidence that differences in vascularisation and metabolic rate could make a significant contribution to the observed differences. Studies on the metabolic activity of various brain structures show that the IC heads the list of brain areas with high metabolic rate both in terms of glucose utilisation (Sokoloff et al., 1977) and blood flow (Landau et al., 1955). In contrast, the MGB is below the average of other auditory areas in these measurements. Given these data on blood flow and energy consumption the pattern of signal differences we observed between IC and MGB is not surprising because the BOLD response is directly linked to changes in blood flow. The question is only whether the metabolic differences between IC and MGB reflect the level of the underlying neuronal responses in the two structures. Alternatively, factors other than neuronal activity must contribute to blood flow changes and glucose consumption in the IC, but this would contradict common assumptions.

Surprisingly, we could not find clear evidence from the electrophysiology literature that would clarify how the IC and the MGB compare in terms of neuronal activity. On the one hand Meeren et al. (2001) report slightly stronger auditory evoked potentials from the IC compared to the MGB recorded from awake and sleeping rats while on the other hand, Suta et al. (2008) recently reviewed and reported generally similar firing rates for single units recorded in the IC and MGB of different species in response to stimuli with different spectro-temporal properties. However, the same review discusses a tendency for more sustained responses to tone bursts in IC compared to much more phasic responses in MGB. The difference is consistent with similar tendencies found for the IC and MGB in human fMRI (Harms and Melcher, 2002, 2003). Due to the long time constant of the haemodynamic response, the BOLD signal integrates the underlying neuronal activity over a period of seconds. Therefore, not only instantaneous spike rate but also the temporal pattern of the neuronal activity is likely to influence the magnitude of the BOLD response. Thus, short phasic neuronal responses could lead to a less robust BOLD signal in the MGB, although this should be reflected in the BOLD response for long stimulus presentation, with a larger initial component, a signature we did not find in our data. At this stage we cannot definitively identify the source of the striking difference in BOLD responses in the IC and MGB. It may be determined by firing rate, but there may also be differences in terms of blood flow and energy consumption that are determined by other factors. Currently, we can only conclude that these differences were consistently observed in our data.

### Activity pattern in the cortex and functional identification of auditory fields

A feature of the activity pattern in the auditory cortex that is particularly noteworthy is the high degree of consistency both across sessions and between the two animals (see Fig. 2, Table 1). For a meaningful comparison of the activity pattern between different animals, the previous identification of individual functional fields based on tonotopy was crucial. The assignment of the observed maxima to the different auditory fields showed that the peak of the cortical responses was always located in the core field A1. In both animals, the activity pattern in A1 featured a medial and a lateral local maximum. With similar consistency a third maximum was found within the borders of the second core field R. Further significantly activated voxels were found in the core field RT and several auditory belt fields, notably the field ML lateral of the field A1. The high degree of agreement between the activity patterns derived from independent experiments across sessions and animals is particularly obvious, because we could co-localise these patterns with functionally defined fields and thus show that activity maxima in specific fields match well between the two animals. In contrast, a reliable assignment of maxima, only millimetres apart, to functionally (or structurally) distinct areas solely based on stereotactic coordinates would have been much more difficult and unreliable.

A general pattern of activity in the auditory cortex with strong responses in the core areas, particularly A1, is in good agreement with previous imaging studies in macaques where simple sound stimuli such as noise or tones were presented (Kayser et al., 2005; Petkov et al., 2006). A few single and multi-unit electrophysiology studies report systematic comparisons of firing rates between core and belt areas. Recanzone (2000) reports slightly higher firing rates in A1 compared to the caudal belt area CM. However, for the clearly stronger response of area A1 compared to R and R compared to RT within the core, there is only weak evidence from electrophysiology. For example, Bendor and Wang (2008) report slightly higher peak firing rates to amplitude-modulated narrow-band stimuli in A1 compared to R and R compared to RT of marmosets. In our study, the difference in response strength between A1 and RT is not only apparent from the large difference in *t*-values (Fig. 2, Table 1), but also in the significantly lower magnitude of the BOLD response (by about 50%) in R in all conditions and in both animals (Fig. 4). A factor that may contribute to an increased response difference between areas in imaging studies is the inherent temporal (and spatial) low pass filter property of the haemodynamic response. As already discussed above, the BOLD response reacts relatively sluggishly and has a limited temporal resolution in comparison to neuronal responses (see also Logothetis, 2002). Similarly, because of the limited spatial resolution of the fMRI method, the BOLD signal we measure integrates the signal of a large number of neurons in one voxel. Thus, the BOLD signal potentially reacts to large-scale effects such as neuronal synchronisation of local neuronal networks that are not obvious in the firing pattern of individual neurons. Fast amplitude modulations which are resolved by single neurons tend to be represented in a unified, strong BOLD response. Based on these predictions, our data fit well with the findings of Bendor and Wang (2008) who report weaker synchronisations in R and RT compared to A1 and Oshurkova et al. (2008) who found more multi-units in A1 compared to the caudal belt field CM that synchronised to amplitude modulated sounds in the range of our study.

Strictly speaking, we cannot determine with certainty whether the different BOLD response magnitudes measured in the various auditory structures are due to differences in neuronal activity in the underlying tissues or due to fundamental differences in their haemodynamic responses or both. Thus, the reported magnitudes have, thus far, mainly descriptive value. However, the outlined procedures for measuring the BOLD signal changes in the auditory pathway of a species that is accessible to intracortical electrophysiology provide the basis for a direct comparison of neuronal activity and the haemodynamic response.

### BOLD response time course comparison within the auditory system

Comparing the shapes of the BOLD signal time courses derived from different stages of the auditory system mainly reveals differences in magnitude rather than general shape. The peaks of the responses are concentrated between 1 or 2 s after sound onset of the 2-s stimulus (Fig. 4, Table 2). In the same time range, we observe a transition to a plateau phase for the BOLD time curves after sustained, 8-s stimulation. Similarly, the signals return to baseline levels at about the same time after stimulus offset for the different auditory regions. Only the BOLD signal from the MGB shows a tendency to exhibit a later absolute maximum, often in the form of a second peak. This second peak, seen in the results of the individual sessions (Fig. 4), could represent an offset peak as shown in the phasic responses of MGB in human auditory fMRI (Harms and Melcher, 2002). However, the second delayed peaks in MGB responses could also simply be explained as an artefact due to generally lower reliability of the BOLD signal from this structure. Higher inter-session variance (also visible in the lower *t*-values measured in the MGB; Table 1), and higher between-session variance (Fig. 4) suggest that the time course from this structure should be interpreted with care.

Taken together, there is no evidence in our data that the BOLD time courses at different levels of the auditory system show substantial differences in shape. This finding is of considerable importance for the comparison of BOLD responses between areas. It implies, for example, that the relative strength of the response is not affected by the exact timing of recording volumes. In contrast to the response shape, there are substantial differences in the response magnitude between the measured areas. These magnitude differences can however be explained by generic differences in the underlying neuronal responses.

### Comparison with time courses of similar experiments in humans and of data from the macaque visual system

The time courses we characterised in this study agree well with time courses established in equivalent experiments conducted in the human auditory cortex (Backes and van Dijk, 2002; Belin et al., 1999). Similar to our experiment, both studies applied a “sparse” design with jittered stimulus onsets to sample the BOLD signal time points at a resolution of 1 or 2 s, respectively. While Backes and van Dijk (2002) triggered the volume acquisition with the subjects heartbeat (HB) with a TRs of 10 HB to achieve cardiac gating, Belin et al. (1999) worked with a fixed repetition time of 10 s. In the experiment by Belin et al. (1999) 600-ms constant harmonic sounds were presented and in Backes and van Dijk (2002) the stimulus was a 700-ms train of white noise bursts.

Both studies reported response peaks between 3 and 4 s after onset of the stimuli which exactly matches the time at which the volume with the maximal BOLD response was recorded after the 2-s stimulus in our study. The maximal percent change in signal of 3% we observed in our study is higher than the data reported in Belin et al. (1999) and Backes and van Dijk (2002). This can likely be attributed to the higher signal-to-noise ratio in our case due to the higher field strength of our magnet and the application of surface coils. In contrast to the study by Belin et al. (1999) that was restricted on the cortical auditory areas, Backes and van Dijk (2002) also reported the BOLD response in brainstem structures. Similar to our study they found no significant difference in the BOLD response time course between brainstem and cortex and they also observed generally lower *t*-values in the MGB than in the IC with only 6 out of 10 subjects showing significant responses in the MGB in contrast to 9 out of 10 in the IC.

In conclusion, there is no evidence from the comparison of our data derived from the macaque auditory cortex with similar data from humans that suggests a fundamentally different haemodynamic response between the two species.

In stark contrast to the similarities across species in the auditory system is the data on the haemodynamic response derived from the visual cortex of the same species (Logothetis, 2002; Logothetis et al., 2001). Logothetis (2002) reports response peaks after about 7–8 s after the onset of 3-s stimulation with checkerboards and about 8 s after the onset of 4-s stimulation (2002; Logothetis et al., 2001). These values are 3–5 s longer, or about twice the times reported in the present study (Fig. 4) and in human auditory studies (Backes and van Dijk, 2002; Belin et al., 1999). Furthermore, we show that the haemodynamic response in our experiment reaches a plateau at 4 s after sound onset of the longer 8-s stimulus (Fig. 4). This compares to a peak at about 10 s after stimulus onset for 6-s visual stimuli and a peak at about 14 s after an onset of 12-s stimulation (Logothetis, 2002).

These considerable differences in the reported time course of the haemodynamic response in two primary sensory areas are surprising. Differences of several seconds can hardly be explained by differences in the underlying neuronal responses in the measured brain areas. While neuronal IC and MGB responses to clicks in non-human primates peak around 5 ms (Velasco et al., 1984) and slightly later for white noise (∼ 12 ms) after stimulus onset (Ryan and Miller, 1977), the auditory cortex shows the first clear neuronal responses 20–50 ms after sound onset, depending on the cortical field and stimulus properties (Bendor and Wang, 2008; Kusmierek and Rauschecker, 2009; Recanzone, 2000). The primary visual cortex V1 of macaques shows very similar response latencies between 30 and 50 ms for black and white gratings similar than in the macaque fMRI studies (Maunsell and Gibson, 1992). Hence, only fundamental differences in the haemodynamics between auditory and visual brain areas, or differences in the way BOLD response has been measured in the studies discussed above could provide a satisfactory explanation for the BOLD time course differences. The slight differences in stimulus duration of the short stimuli do not provide a conclusive explanation. The duration differences are too small, and if a linear stimulus-response relationship is assumed, peak differences larger than stimulus duration differences are not expected. Furthermore, Logothetis et al. (2001) also provide a calculation of the impulse response (the response to a theoretical infinitesimally short stimulus) with peak values at 6–7 s after stimulus onset. Although, in line with previous findings in humans (Birn et al., 2001; Boynton et al., 1996), a linearity examination showed that assuming linearity would overestimate the magnitude of the BOLD response to a longer stimulus predicted from a shorter one, the latency of the peak or plateau onset is hardly affected (Fig. 4C). If anything, the measured nonlinearities would predict even shorter peak latencies for longer stimuli than would be expected assuming a linear behaviour. Thus, the observed nonlinearity effects cannot explain the disparity between the findings for the two sensory systems.

It is worth mentioning that in contrast to our study, the studies on the macaque visual system used anaesthetised animals. A recent study from the same group (Goense and Logothetis, 2008) with awake animals seems to show slightly earlier BOLD signal peaks but the study is not detailed enough on that matter to allow a conclusion. However, studies on the visual system in awake humans also report relatively late peaks in the range reported by Logothetis et al. (2002; Logothetis et al., 2001) in response to checkerboard presentations (e.g., Boynton et al., 1996).

Finally, the applied MRI parameters could be a factor contributing to the differences between the studies. One factor the auditory studies have in common is that they are conducted with a sparse design – the only auditory design that avoids recording the BOLD response in a saturated state due to the scanner noise – and as a consequence of the very long TR, full relaxation of the T1 magnetisation is reached. This allows the application of higher flip angles of typically 90° (this study; Backes and van Dijk, 2002; Belin et al., 1999) to achieve enhanced signal strength. Flip angle, TR and level of relaxation can shift the balance of contribution between capillaries, small and large blood vessels due to inflow effects (Frahm et al., 1994; Haacke et al., 1994). Thus, it cannot be excluded that an emphasis on different types of blood vessels with different haemodynamics contributes to the differences in the reported time courses of the BOLD signal between auditory and visual areas. To address this question, comparisons of auditory and visual BOLD responses recorded with identical MRI parameters, ideally in the same experiment, are required.

## Conclusion

In this study we characterised the time course of the BOLD signal to salient auditory stimuli in subcortical and cortical structures of the auditory pathway in macaques. Owing to the use of awake monkeys and due the applied sparse design, the measured BOLD response is unaffected by anaesthesia or scanner noise and can be directly compared to human fMRI results. The comparison of the different time courses for structures within the auditory pathway showed a common temporal pattern but systematic differences in the response magnitudes with maximal values in A1. The temporal pattern established in our monkeys agrees well with the data recorded in similar human fMRI experiments. However, previous studies from the visual cortex in macaques report considerably later BOLD response peaks.

The detailed time courses characterised in this study form a body of reference data that facilitate the optimal design of future continuous and sparse fMRI studies in the auditory system of non-human primates. Nevertheless, the poor match of the temporal BOLD response pattern between studies from auditory and visual areas demonstrates that caution is necessary when designing an fMRI experiment based on the time course of the BOLD response measured from different brain areas with different MRI parameters.

## Figures and Tables

**Table 1:** Local maxima in t-maps for individual sessions (in *t*-values).

Session	Monkey C (2-s stimulus)						Monkey C (8-s stimulus)					
	I	II	III	IV	V	Mean	I	II	III	IV	V	Mean
A1	9.21	14.63	16.9	13.54	11.64	13.18	21.93	11.67	24.65	17.91	26.54	20.54
R	7.32	10.71	13.99	9.79	7.2	9.80	15.08	10.07	14.94	10.3	15.27	13.13
MGB	3	6.54	5.91	9.1	8.21	6.55	6.73	8.34	7.15	6.72	11.22	8.03
IC	4.75	7.27	8.45	8.41	8.43	7.46	11.86	11.33	12.66	13.56	18.34	13.55


Session	Monkey W (2-s stimulus)						Monkey W (8-s stimulus)					
	I	II	III	IV	V	Mean	I	II	III	IV	V	Mean
A1	12.4	6.57	6.78	7.67	3.47	7.38	10.77	7.48	10.23	9.48	10.43	9.68
R	10.63	5.93	4.87	5.78	1.91	5.82	6.85	3.46	5.3	3.25	4.16	4.60
MGB	7.71	4.36	2.65	3.66	4.08	4.49	6.46	3.52	5.6	5.19	8.52	5.86
IC	7.68	6.72	5.3	7.37	8.66	7.15	9.86	9.39	8.96	7.62	22.53	11.67

**Table 2:** BOLD response peaks (2-s stimulus).

	Area	Signal change [%]		Peak latency [s]	
		Mean	std	Mean	std
Monkey C (*n* = 5)	A1	2.79	0.18	4.00	-
R	1.73	0.58	4.40	0.55
IC	2.06	0.44	4.40	0.89
MGB	2.58	0.51	4.80	0.45
		A1 > R	*p* = 0.0044⁎⁎	MGB > A1 *p* = 0.0039⁎⁎	
		A1 > IC	*p* = 0.0087⁎⁎		
		MGB > R	*p* = 0.038⁎		
Monkey W (*n* = 5)	A1	2.63	0.57	3.80	0.45
R	1.26	0.18	4.00	0.71
IC	2.31	0.25	3.80	0.45
MGB	1.67	0.68	4.40	1.52
		A1 > R	*p* < 0.001⁎⁎⁎		
		A1 > MGB	*p* = 0.043⁎		
		IC > R	*p* < 0.001⁎⁎⁎		

⁎ *p* < 0.05.

⁎⁎ *p* < 0.01.

⁎⁎⁎ *p* < 0.001. std: standard deviation.
